# It does not always take two to tango: “Syntrophy” *via* hydrogen cycling in one bacterial cell

**DOI:** 10.1038/s41396-020-0627-1

**Published:** 2020-03-16

**Authors:** Anja Wiechmann, Sarah Ciurus, Florian Oswald, Vinca N. Seiler, Volker Müller

**Affiliations:** Molecular Microbiology & Bioenergetics, Institute of Molecular Biosciences, Johann Wolfgang Goethe University, Frankfurt am Main, Germany

**Keywords:** Microbial ecology, Bacterial genetics, Bacterial physiology

## Abstract

Interspecies hydrogen transfer in anoxic ecosystems is essential for the complete microbial breakdown of organic matter to methane. Acetogenic bacteria are key players in anaerobic food webs and have been considered as prime candidates for hydrogen cycling. We have tested this hypothesis by mutational analysis of the hydrogenase in the model acetogen *Acetobacterium woodii*. Hydrogenase-deletion mutants no longer grew on H_2_ + CO_2_ or organic substrates such as fructose, lactate, or ethanol. Heterotrophic growth could be restored by addition of molecular hydrogen to the culture, indicating that hydrogen is an intermediate in heterotrophic growth. Indeed, hydrogen production from fructose was detected in a stirred-tank reactor. The mutant grew well on organic substrates plus caffeate, an alternative electron acceptor that does not require molecular hydrogen but NADH as reductant. These data are consistent with the notion that molecular hydrogen is produced from organic substrates and then used as reductant for CO_2_ reduction. Surprisingly, hydrogen cycling in *A. woodii* is different from the known modes of interspecies or intraspecies hydrogen cycling. Our data are consistent with a novel type of hydrogen cycling that connects an oxidative and reductive metabolic module in one bacterial cell, “intracellular syntrophy.”

## Introduction

Molecular hydrogen is present only in trace concentrations (550 parts per billion) in the Earth’s atmosphere [1], but plays an important part in the global carbon cycle and is a major constituent of microbial metabolism. In anoxic ecosystems it is rapidly produced and consumed by microorganisms resulting in a large turnover [2]. Hydrogen connects different parts of the anaerobic food web and is usually produced by primary fermenters [3]. Fermentations typically yield between 1 and 4 mol of ATP per mol of sugar, and the maximum is only observed if electrons can be blown away into the environment as molecular hydrogen thus allowing the cells to make acetate according to Eq. (1) [4]:

1 $${\rm{C}}_{6}{\rm{H}}_{12}{\rm{O}}_{6} +	 {\rm{2H}}_{2}{\rm{O}} + {\rm{4ADP}} + {\rm{4P}}_{\rm{i}}{\longrightarrow}{\rm{2CH}}_{3}{\rm{COOH}} \\ +	 {\rm{2CO}}_{2} + {\rm{4H}}_{2} + {\rm{4ATP}} \,\,\, \Delta{\rm{G}}^{0\prime} = -206.3 \,\,{\rm{{kJ/mol}}}$$

However, hydrogen formation from reduced pyridine nucleotides or flavins is energetically unfavourable and growth according to Eq. (1) requires removal of hydrogen by a syntrophic partner such as a sulfate reducing bacterium, a methanogenic archaeon or an acetogenic bacterium [5–8]. The latter produces acetate according to Eq. (2):

2 $$4{\rm{H}}_{2} +	 {\rm{2CO}}_{2} + {\rm{xADP}} + {\rm{xP}}_{\rm{i}}{\longrightarrow} {\rm{CH}}_{3}{\rm{COOH}} \\ +	 {\rm{2H}}_{2}{\rm{O}} + {\rm{xATP}}\,\,\,\Delta{\rm{G}}^{0\prime} = -95 \,\,{\rm{{kJ/mol}}}$$

Since acetogens grow by conversion of H_2_ + CO_2_ to acetate, the reaction has to be coupled to net synthesis of ATP [9]. Detailed studies in the acetogenic model organism *Acetobacterium woodii* estimated the amount of ATP to 0.3 mol per mol of acetate produced [9].

**Fig. 1:**
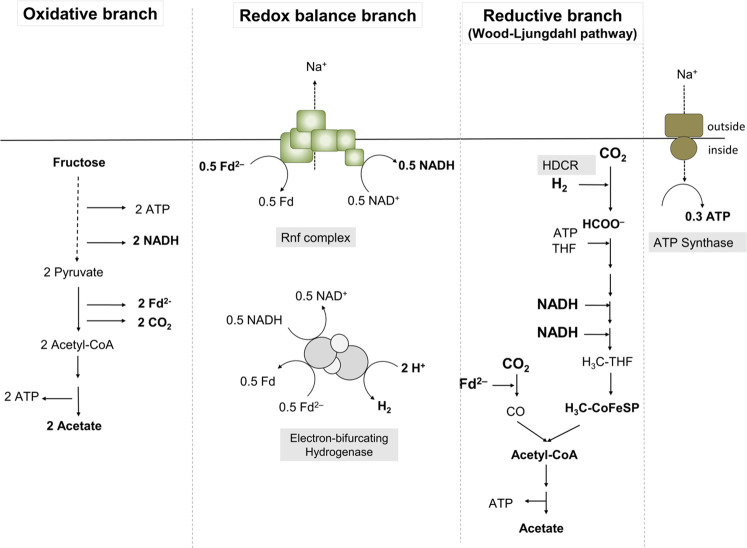
The modularity of acetogenesis in *A. woodii*. Shown are the oxidation of fructose to acetate in the oxidative branch (left) and the reduction of CO_2_ to acetate (right) in the reductive branch (WLP). Redox balancing is achieved by a third module, in which the Rnf complex and the electron-bifurcating hydrogenase produce the reductants required for the the WLP. Fd, ferredoxin; Fd^2−^, reduced ferredoxin; THF, tetrahydrofolate; HDCR, hydrogen-dependent CO_2_ reductase; CODH/ACS, carbon monoxide dehydrogenase/acetyl-CoA synthetase; Co-FeS-P, corronoid iron-sulfur protein.

In contrast to methanogenic archaea, acetogenic bacteria do not only grow lithotrophically according to Eq. (2) but also by fermentation [10]. Acetogenesis is a modular metabolism with an oxidative and a reductive branch [11] (Fig. 1). In the oxidative branch, hydrogen (during lithotrophic growth) or an organic carbon and energy source (during heterotrophic growth) are oxidised. Electrons are carried over to the reductive branch (the Wood-Ljungdahl pathway [WLP]) in which 2 mol of CO_2_ are reduced to acetate according to Eq. (2). Overall, fermentation of fructose to three molecules of acetate by a combination of Eqs. (1) and (2) gives the highest ATP yield in fermenting bacteria of 4.3 mol ATP/mol of sugar [11].

Electron carriers involved in transferring electrons from the oxidative to the reductive branch in acetogens are NADH, NADPH, or reduced ferredoxin [9]. A third module, the redox balance module, ensures that the different reduced electron carriers from the oxidative module are fed in and are converted to the specific redox carriers required by the WLP [11]. In the model acetogen *A. woodii*, 2 mol of NADH from glycolysis and 2 mol of reduced ferredoxin from conversion of pyruvate to acetyl-CoA are converted to 1 mol of hydrogen, 2 mol of NADH and 1 mol of reduced ferredoxin by the combined action of the Rnf complex and the electron-bifurcating hydrogenase [11, 12]. NADH is used as reductant for the methylene-tetrahydrofolate (THF) reductase [13] and the methylene-THF dehydrogenase [14], and reduced ferredoxin is used by the CO dehydrogenase/acetyl-CoA synthase as electron donor for CO_2_ reduction in the carbonyl branch of the WLP [15, 16]. In contrast, the first enzyme used for CO_2_ reduction in the methyl branch of *A. woodii* (Fig. 1), the hydrogen-dependent CO_2_ reductase (HDCR), uses H_2_ as reductant in vitro [17], indicating the need for electron transfer *via* hydrogen from the oxidative module (glycolysis) to the reductive module (CO_2_ reduction). On the other hand, the purified HDCR can also accept electrons from reduced ferredoxin, albeit with 17-fold lower activities [17]. To address a potential hydrogen transfer from the oxidative to the reductive branch of the acetogenic metabolism, we have deleted the only hydrogenase in *A. woodii* and studied the phenotype of the mutant.

## Materials and methods

### Growth of *A. woodii*

*A. woodii* DSM1030 was cultivated at 30 °C under anoxic conditions in complex medium as previously described [18]. When using the *pyrE* deletion mutant, 50 mg/l uracil was added to the medium [12]. Unless otherwise stated 20 mM fructose or 20 mM fructose together with H_2_ + CO_2_ (80:20 [v/v]) were used as carbon source. Gaseous substrates were used at a pressure of 1.0 × 10^5^ Pa. For growth experiments, concentrations of the used carbon sources were as follows: lactate, 80 mM; ethanol, 50 mM; formate, 100 mM. Minimal medium used for genetic manipulations was prepared as previously described [19] in which yeast extract was omitted and higher amounts of 0.2 g l^−1^ KH_2_PO_4_, 1.35 g l^−1^ NH_4_Cl, and 1.5 ml l^−1^ of selenite/tungstate solution were used and 10 µg ml^−1^ of D/L-pantothenate was added. For mutant selection, 1.7 µg ml^−1^ uracil and 1.5 mg ml^−1^ 5-fluoroorotic acid were added.

### Genetic modifications

For deletion of the *hydBA* genes, an uracil auxotroph *pyrE* deletion mutant was generated using the suicide plasmid pMTL_AW_KO1. The background of the plasmid pMTL_AW_KO1 is pMTL84151 [20] out of which the Gram^+^ origin of replication was partially deleted by digestion of the vector with *Xmn*I and *Fsp*I following a blunt-end ligation. The *pyrE* deletion cassette was cloned into the multiple cloning site, consisting of a 393 bp upstream flanking region including the first 52 bp of *pyrE* and a 399 bp downstream flanking region including the last 68 bp of *pyrE*. Both flanking regions were amplified *via* PCR, joined by splice-by-overlap-PCR (SOE-PCR) and cloned into the plasmid using *Bam*HI and *Eco*RI. Transformation and integration of the plasmid into the *A. woodii* wild type as well as recombination of the plasmid at its homologous regions toward the loss of the *pyrE* gene has already been described elsewhere [12]. The deletion of 456 bp of the *pyrE* gene was verified by DNA sequencing analysis [21]. The suicide plasmid pMTL_AW_KO2 for the in-frame deletion of *hydBA* genes in *A. woodii* was built in the pMTL_AW_KO1 background. First, the *pyrE* cassette from *Eubacterium limosum* KIST612, consisting of the *pyrE* gene (ELI_0961) and 66 bp of its promoter region, was placed behind the *catP* resistence marker cassette to be used as a counter selectable marker as described previously [12], generating pMTL_AW_KO1_*pyrE*_Elim. Second, both fragments of the *hydBA* deletion cassette, consisting of a 486 bp upstream flanking region including the first 18 bp of *hydB* and a 462 bp downstream flanking region including the last 15 bp of *hydBA*, where amplified* via* PCR, ligated *via* SOE-PCR and were cloned into pMTL_AW_KO1_*pyrE*_Elim by using *Eco*RI and *Xba*I, which replaced the original *pyrE* deletion cassette with the *hydBA* deletion cassette. For plasmid transformation into the *pyrE* mutant and further integration and recombination of the *hydBA* deletion cassette, the same protocol as for the *pyrE* gene deletion, as described above, was followed. The deletion of 3538 bp of the *hydBA* region was verified by DNA sequencing analysis [21].

### Preparation of cell-free extracts

Cells were harvested in the late exponential growth phase and resuspended in lysis buffer, containing 25 mM Tris-HCl buffer, pH 7.8, 420 mM sucrose, 2 mM DTE, 4 µM resazurin, for a 1 h treatment with 2.8 mg ml^−1^ lysozyme. After washing the protoplasts in analytical buffer, containing 25 mM Tris-HCl, pH 7.6, 20 mM MgSO_4_, 20 % [v/v] glycerol, 2 mM DTE, 4 µM resazurine, 0.5 mM PMSF, 0.1 mg ml^−1^ DNAseI, protoplasts were passed twice through a french pressure cell at 110 MPa (Thermo, Needham Hights, MA, USA). The cell-free extract was separated from cell debris by centrifugation at 12,000 × *g* for 15 min.

### Hydrogenase activity assays

The activity of the electron-bifurcating hydrogenase was determined as described previously [22].

### Western blot analysis

For detection of HydB and HydA subunits in cell-free extracs, Western blot analysis was performed as described before [23].

### Stirred-tank reactor cultivations

Bioreactor cultivations were conducted in a 2 l working volume Biostat Aplus fermenter (Sartorius, Melsungen, Germany). The vessel was equipped with temperature probe, sparger, baffles, two Rushton-impeller, pH-probe (Hamilton, Bonaduz, Switzerland) and a redox potential probe (Hamilton, Bonaduz, Switzerland). The gas stream into the reactor was maintained at a constant rate by a digital mass-flow controller (Bronkhorst High-Tech, Ruurlo, Netherlands).

### Analytical methods

Protein concentrations were determined by the method described by Bradford [24]. Fructose, formate and acetate concentrations were measured enzymatically (Hoffmann-La Roche, Basel, Switzerland). Hydrogen concentrations were determined by gas chromatography (GC) as described previously [17, 25]. Fermentation off-gas analysis was conducted by a Micro-GC (Inficon, Bad Ragaz, Switzerland) equipped with two measurement modules. Module one had argon as carrier gas for determination of hydrogen, oxygen and nitrogen, module two had helium as carrier gas for determination of carbon dioxide and water. Sampling time of the GC was 25 s. The analytical conditions for module one were: injector temperature, 90 °C; column pressure, 2.07 × 105 Pa; column temperature, 80 °C, column, Rt-Molsive 5 Å, 0.25 mm ×10 m with a Rt-Q-Bond, 3 m precolumn and for module two: injector temperature, 90 °C; column pressure, 1.72 × 105 Pa; column temperature, 60 °C, column, Rt-Q-Bond, 0.25 mm × 8 m. Each module was equipped with a thermal conductivity detector and the sampling rate was set to 100 Hz. Analysis time was 90 s for both modules. Off-line samples were taken in 2 ml volume. OD_600_ was measured in a spectrophotometer, Genesys 10 s UV-Vis (Thermo Scientific, Madison, USA) at a wavelenth of 600 nm. Samples were diluted with 0.9 % NaCl solution, if necessary. Samples were centrifuged at 16,363 × *g* for 10 min and the supernatant was used for analysis of acetic acid, formic acid and fructose using enzymatic assays as described above.

## Results

### Deletion of the *hydBA* genes of the hydrogenase operon (*hydCEDBA*)

**Fig. 2:**
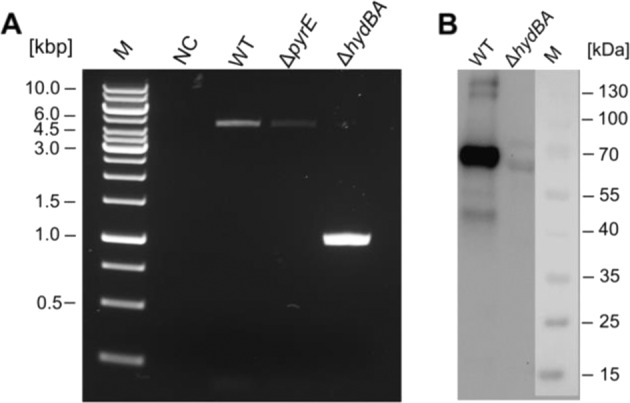
Verfication of the *hydBA* deletion in *A. woodii* Δ*pyrEvia* PCR and Western Blot analysis. Agarose gel showing PCR products of the wild type (WT), *pyrE* (Δ*pyrE*) and *hydBA* mutant (Δ*hydBA*) using primers which anneal outside the *hydBA* region (**a**). Detection of HydB and HydA in crude extract of the wild type and *hydBA* mutant blotted on a nitrocellulose membrane and by using antibodies against HydB and HydA (**b**). M, Marker; NC, negative control; WT, *A. woodii* wild type.

Apart from the hydrogenase module in the HDCR, *A. woodii* has only one hydrogenase (HydABCD), a soluble, electron-bifurcating enzyme [22]. To delete the genes coding for the two major subunits HydB (Awo_c26980) and HydA (Awo_c26970) of the electron-bifurcating hydrogenase encoded by the *hydCEDBA* operon (Awo_c27010-Awo_c26970), the suicide plasmid pMTL_AW_KO2 was generated. The plasmid contains homologous flanking regions of ~480 bp upstream and downstream of the *hydBA* genes each leaving 15 bp plus the start codon of *hydB* and 12 bp plus the stop codon of *hydA* intact after deletion. The suicide plasmid was integrated into the chromosome at one of the flanking regions under antibiotic pressure. The following desintegration was forced by the presence of 5-fluoroorotate since the plasmid contains a *pyrE* gene together with its promoter for production of a functioning orotate phosphoribosyltransferase. However, isolation of mutants using fructose as only carbon source failed. Since this may have been caused by the lack of molecular hydrogen produced by HydABCD, hydrogen was added to the culture. Indeed, colonies were obtained, isolated and characterised. PCR (Fig. 2a) and DNA sequencing analyses confirmed the absence of *hydBA* in the genome. HydB and HydA could not be detected with antibodies against the two subunits (Fig. 2b) and cell-free extract of these cells did not catalyse hydrogen-dependent NAD^+^ and ferredoxin reduction. These experiments demonstrate that *hydBA* were deleted.

### Hydrogen restores growth of the *hydBA* mutant on organic substrates

That growth of the *hydBA* mutant on fructose is dependent on the addition of hydrogen is in line with the hypothesis that hydrogen is an electron carrier between the oxidative and reductive branch of acetogenesis (Fig. 1). This is also supported by the observation that the mutant did not grow on lactate or ethanol without addition of hydrogen. Formate is an intermediate of the WLP, and is dismutated to CO_2_ and acetate. As expected, the *hydBA* mutant did not grow on formate and addition of hydrogen also did not restore growth of the mutant (Table 1). Formate oxidation to CO_2_ yields hydrogen and obviously, the hydrogenase is essential to reduce NAD and ferredoxin, two electron carriers essential for CO_2_ reduction, with H_2_ as reductant.

**Table 1:** Growth of the *A. woodii* wild type and the *hydBA* mutant in bicarbonate-supplied medium supplemented with different carbon sources.

	Without H_2_		Addition of H_2_	
	Wild type	*hydBA* mutant	Wild type	*hydBA* mutant
**Fructose**	+	−	+	+
**Lactate**	+	−	+	+
**Ethanol**	+	−	+	+
**2,3-Butanediol**	+	−	+	+
**Formate**	+	−	+	−

The addition of hydrogen (H_2_ + CO_2_, 80:20 [v/v], at 1.0 × 10^5^ Pa) is indicated. Growth was observed, whereby a plus symbol (+) indicates growth and a minus symbol (−) indicates no growth.

### Hydrogen transfer in *A. woodii*

**Fig. 3:**
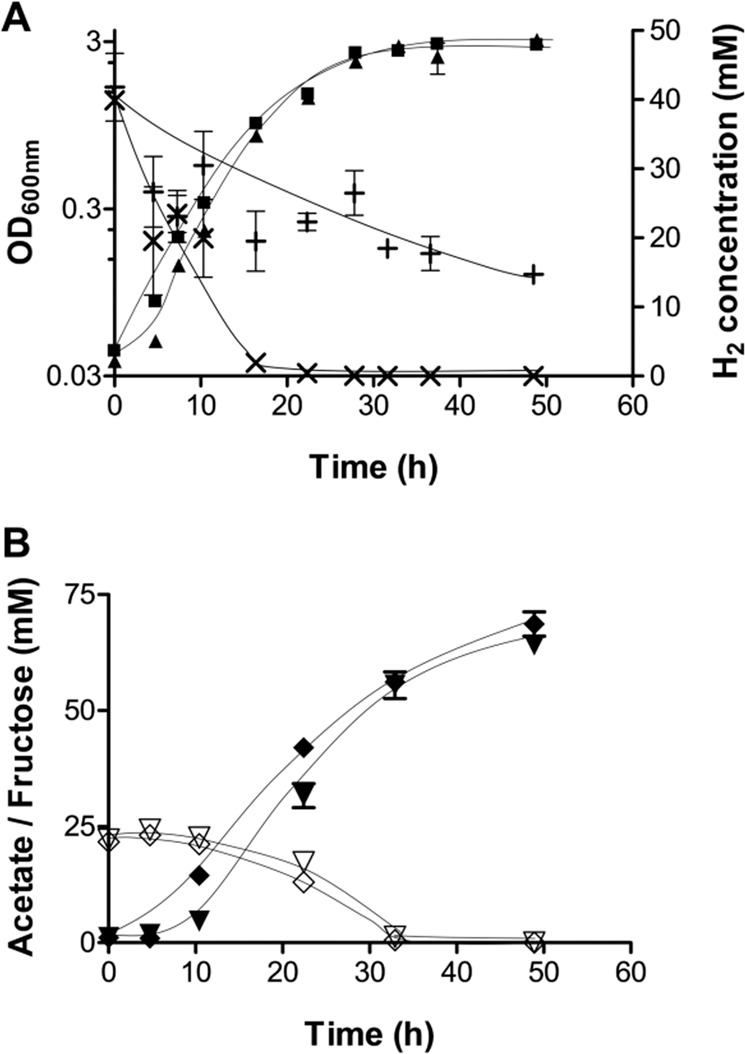
Growth and metabolite concentrations in *A*. *woodii* wild type and *hydBA* mutant in bicarbonate-supplied medium supplemented with 20 mM fructose + hydrogen. Displayed are the optical densities of the wild type (■) and the *hydBA* mutant (▲) as well as the hydrogen concentration in the headspace of the wild type (×) and the *hydBA* mutant (+) cultures (**a**). The fructose consumption of the wild type (◇) and *hydBA* mutant (▽) together with the concomitant production of acetate in the wild type (◆) and *hydBA* (▼) mutant was monitored over time (**b**). (*n* = 2).

Next, we tested for hydrogen consumption and production during growth of the mutant and the wild type in bicarbonate-supplied medium supplemented with 20 mM fructose under an H_2_ + CO_2_ (80:20 [v/v]) atmosphere. In theory, 1 mol of hydrogen should be produced by the electron-bifurcating hydrogenase per mol of fructose consumed, which then should be used by the HDCR for CO_2_ reduction (Fig. 1). The growth rate of wild type and mutant (Fig. 3a) was similar (each ~0.16 h^−1^) as well as the acetate:fructose ratio of 3:1 in the stationary phase (Fig. 3b), but the mutant cells used only ~20 mM of hydrogen (and 20 mM fructose), in contrast to the wild type which consumed all the hydrogen (Fig. 3a). This result confirms that the HDCR requires hydrogen and that hydrogen is produced by the electron-bifurcating hydrogenase when grown on substrates such as fructose, lactate or ethanol.

### Initial stirred-tank reactor (STR) cultivation of the *A. woodii* wild type revealed in vivo hydrogen formation

**Fig. 4:**
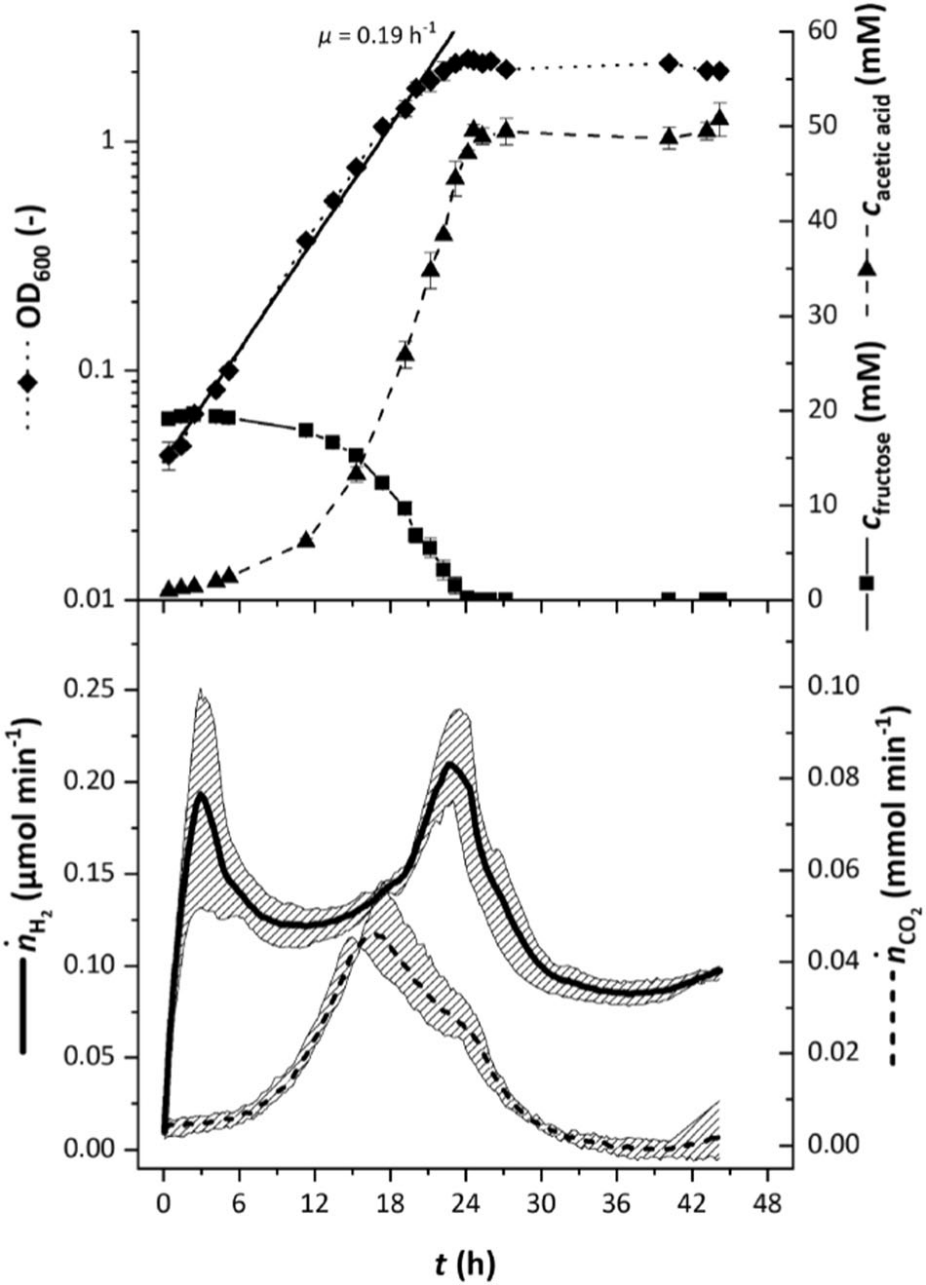
Off-line and off-gas data for stirred-tank reactor fermentations of *A. woodii* wild type. The wild type was grown in complex medium with 20 mM fructose as carbon and energy source and the cultivation broth was sparged with 10 ml min^−1^ 20 % CO_2_ in nitrogen. The upper half shows the average off-line values for OD_600_ (◆), concentration of fructose (■) and acetic acid (▲) of four cultivations whilst the bottom half shows off-gas flow rates for hydrogen (solid line) and carbon dioxide (dashed line). The grey areas in the bottom half depict the variation of the off-gas values.

If hydrogen is produced in vivo to connect the oxidative and reductive parts of acetogenesis, then, due to its volatile nature, it should be detectable in the gaseous phase. To determine a possible hydrogen production, a bench-scale anaerobic bioreactor for cell cultivation was set up together with off-gas analysis *via* GC. Initial cultivations of the *A. woodii* wild type in bicarbonate-supplied medium with 20 mM fructose as carbon and energy source showed exponentially rising hydrogen flow rates in the off-gas of up to 0.24 µmol min^−1^ (Fig. 4). Hydrogen levels in the off-gas dropped to 0.1 µmol min^−1^ after fructose was completely consumed. The cultures reached a maximum OD_600_ of 2.28 after 24 h of cultivation, the growth rate was 0.19 h^−1^. From the available 20 mM fructose, 50 mM of acetate was produced after 24 h. Overall 2.4 mol of acetic acid and 8.4 ± 0.2 mmol of hydrogen were produced per mol of fructose consumed. *A. woodii* requires Na^+^ [18] and a functional Rnf complex for energy conservation [12]. When grown with fructose in the absence of Na^+^, the acetate:fructose ratio of the wild type decreased to 2 and 2.1-times more hydrogen was released. Similar results were observed for the *rnf* deletion mutant. Maximum values for OD_600_ and growth rate were 1.85 and 0.13 h^−1^ for the *rnf* mutant and 1.68 and 0.11 h^−1^ for the wild type (data not shown). Table 2 summarises the above mentioned fermentation parameters.

**Table 2:** Selected fermentation parameters from STR-cultivations of the *A*. *woodii* wild type (± Na^+^) and the *rnf* mutant.

Parameter	WT + Na^+^	WT −Na^+^	*rnf* mutant
OD_600, max_/−	2.28 ± 0.06	1.66 ± 0.01	1.85 ± 0.01
*µ*/h^−1^	0.19	0.11	0.13
*Ỹ*_P/S_/mol mol^−1^	2.44 ± 0.02	2.01 ± 0.05	2.06 ± 0.03
*Ỹ*_H2/S_/mmol mol^−1^	8.39 ± 0.25	18.74 ± 0.62	17.81 ± 1.84

OD_600, max_, maximum OD; *µ*, growth rate; *Ỹ*_P/S_, yield of acetic acid per fructose; ${\tilde {Y}_{{\mathrm{{H}}}_2/S}}$, yield of hydrogen per fructose; WT, wild type.

### Replacing the WLP with other, non-hydrogen consuming reductive pathways restores growth of the *hydBA* mutant on fructose

**Fig. 5:**
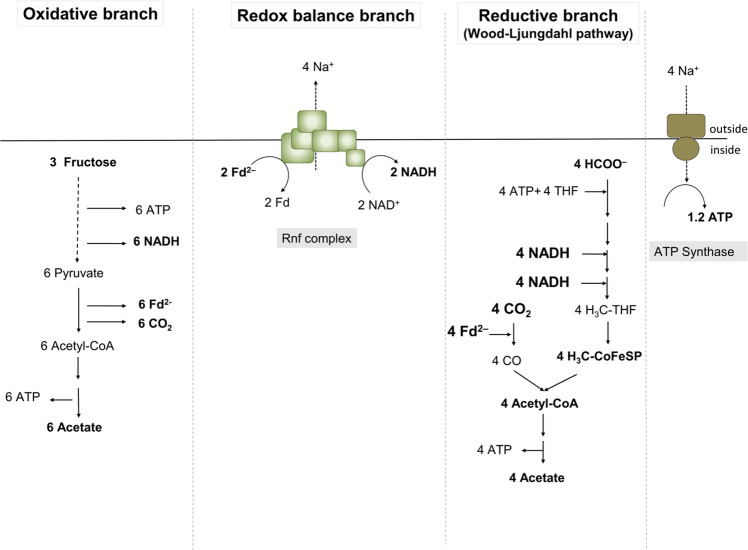
Schematic overview of fructose metabolism of *A*. *woodii* using formate instead of CO_2_ as electron acceptor in the reductive branch. Fd, ferredoxin; Fd^2−^, reduced ferredoxin.

H_2_ + CO_2_ is converted to formate by the HDCR [17]. Therefore, we speculated that the *hydBA* mutant should be able to grow on fructose + formate (+ CO_2_) instead of fructose + CO_2_, since formate reduction only requires NADH and reduced ferredoxin (Fig. 5). Indeed, the *hydBA* mutant grew with a doubling time of ~5 h on 20 mM fructose and 100 mM formate, which is slightly slower than the growth of the wild type (~3.5 h–4 h). The final OD_600_ of the *hydBA* mutant reached 80–85 % of the final OD_600_ of the wild type.

**Fig. 6:**
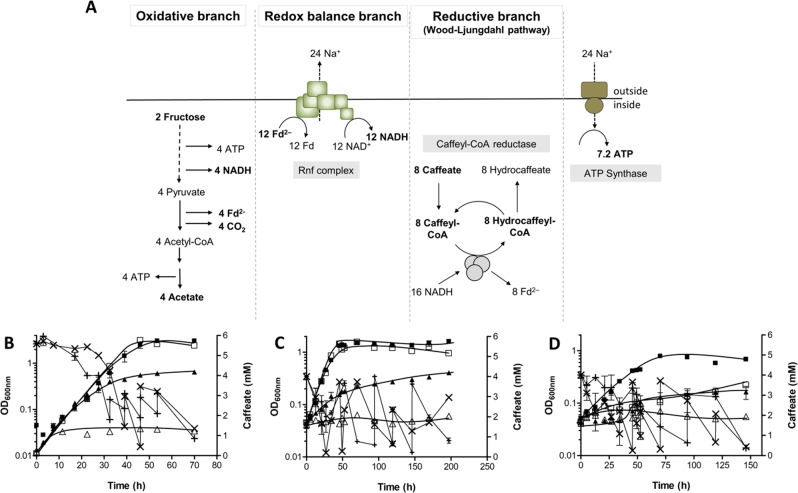
Schematic overview of the fructose metabolism of *A*. *woodii* using caffeate as final electron acceptor and growth of *A. woodii* wild type and the *hydBA* mutant on different carbon sources with caffeate as electron acceptor. Fd, ferredoxin; Fd^2−^, reduced ferredoxin (**a**). Cells were grown in complex medium supplemented with either 20 mM fructose + 6 mM caffeate (**b**), 80 mM lactate + 4 mM caffeate (**c**) or 50 mM ethanol + 4 mM caffeate (**d**). Growth of the wild type + caffeate (■), *hydBA* mutant + caffeate (▲), wild type without caffeate (□), *hydBA* mutant without caffeate (△) were measured over time at 600 nm. Caffeate was added when concentrations dropped below 1 mM and its utilisation by the wild type (×) and *hydBA* mutant  (+) was monitored over time (*n* = 2).

*A. woodi* can use caffeate as electron acceptor alternative to CO_2_ [26, 27]. Reduction of caffeate to hydrocaffeate does not require hydrogen but only NADH as reductant [28] (Fig. 6a). Therefore, the *hydBA* mutant should be able to grow on, for example, fructose + caffeate. After addition of fructose + caffeate to the wild type and the mutant, growth of the two strains could be detected (Fig. 6b). Doubling times of the mutant and the wild type were similar (~6 h), but growth of the mutant culture stopped much earlier, with a final OD_600_ of 0.68 compared with 3.2 of the wild type. The mutant also grew on lactate + caffeate (Fig. 6c) or ethanol + caffeate (Fig. 6d), but the growth rate of the *hydBA* mutant was one fifth and one half of the growth rate of the wild type, respectively. Like for fructose + caffeate, the final OD_600_ was 0.47 or 0.25 for the mutant and 1.5 or 0.78 for the wild type when grown on lactate + caffeate or ethanol + caffeate, respectively. The lower final OD_600_ may be due to the inability of the strains to use the HDCR reaction for recapturing CO_2_ which is produced during the oxidation of the respective substrate.

## Discussion

Sulfate reducing bacteria grow by oxidation of lactate or other organic substrates coupled to energy conservation by the reduction of sulfate [29, 30]. They also grow lithotrophically and, indeed, hydrogen is the most effective hydrogen donor for sulfate reduction [4, 29]. This led Odom and Peck to postulate hydrogen cycling as a general mechanism for energy coupling in sulfate reducing bacteria [31] and they postulated this mechanism to be present in methanogens and acetogens as well [5]. According to their model, hydrogen is produced inside the cell and diffuses across the membrane to the periplasm. The authors speculated that the same organism oxidises hydrogen to 2 H^+^ + 2e^−^ at the periplasmic side of the membrane, thus, producing scalar protons that create a proton motive force (pmf) across the cytoplasmic membrane that drives ATP synthesis. This mechanism requires a soluble, cytoplasmic hydrogenase and a periplasmic, membrane-bound hydrogenase. Biochemical and physiological experiments performed are in line with this hypothesis [32]. More than 35 years later, hydrogen cycling as a mode of pmf generation was directly demonstrated by mutational analyses for the archaeon *Methanosarcina barkeri* [33]. When grown on methanol, 25 % of the substrate is oxidised to CO_2_ to generate the electrons needed to reduce the other 75 % to methane. The oxidation of methanol is coupled to the reduction of protons to H_2_, as catalysed by the F_420_-reducing hydrogenase. Hydrogen diffuses out of the cell and is harnessed by the membrane-bound Vht hydrogenase that reduces the membrane-integral electron-carrier methanophenazine, the electron donor for the respiratory enzyme, the heterodisulfide reductase [33]. The proton potential established by the heterodisulfide reductase drives the synthesis of ATP in this archaeon [34]. Deletion of the soluble hydrogenase abolished hydrogen formation and deletion of the Vht hydrogenase was lethal, demonstrating nicely that there is hydrogen cycling in one cell, important for energy conservation [33].

The second type of hydrogen transfer in anoxic ecosystems is observed between different species and is called interspecies hydrogen cycling [5]. There, one partner oxidises a substrate linked to the production of hydrogen. Since hydrogen formation is thermodynamically unfavourable, the hydrogen producer can only survive if the hydrogen concentration in the environment is kept low by hydrogen oxidising microorganisms such as methanogenic archaea [6, 7].

Whether or not hydrogen cycling occurs in the ecophysiologically relevant group of acetogenic bacteria remained to be established. In this report, we show clear evidence that *A. woodii* releases hydrogen into the atmosphere but only ~8.4 mmol hydrogen per mol of fructose was liberated, which is less than 10 % of the acetic acid generated, but similar to the result of Braun et al. [35]. This result indicates that the HDCR efficiently captures the hydrogen produced by the electron-bifurcating hydrogenase. If the electron-bifurcating hydrogenase is missing, cells growing on fructose, ethanol or lactate are unable to produce hydrogen needed for CO_2_ reduction by the HDRC and therefore are unable to grow, except when they grow mixotrophically on fructose + hydrogen or fructose + formate. This is also clear evidence that hydrogen evolution takes place inside the cell and hydrogen is directly used within the cell before it can diffuse through the cell membrane. Since *A. woodii* lacks a membrane-bound hydrogenase, hydrogen oxidation is not linked to energy conservation and, therefore, does not fall into the category “intraspecies hydrogen cycling linked to energy conservation”, as postulated by Odom and Peck [5].

In contrast, *A. woodii* combines the metabolic features of two syntrophic partners in one bacterial cell. Depending on the environmental conditions *A. woodii* can play the part of the fermenting partner as in coculture with a methanogen [36] or the hydrogen consuming partner in syntrophic interactions. When grown together with methanogens on H_2_ + CO_2_, acetogens usually would be outcompeted by methanogens since methanogenesis from H_2_ + CO_2_ delivers much more energy than acetogenesis [37]. However, under certain conditions acetogens dominate as H_2_ + CO_2_ -consuming partner in syntrophic interactions. For example, hydrogen-utilising acetogens compete successfully with hydrogen-utilising methanogens in wood-feeding termites [38]. Further studies showed that acetogens can outcompete methanogens for hydrogen at a pH of 6.2 and also at more acidic pH values [39] and at low temperature, for example at an in situ temperature of 4 °C in sediments of Lake Constance [40]. The partial pressure of hydrogen measured in pore water is too low to allow growth of pure cultures and it is speculated that the in situ partial pressure of hydrogen might be higher for acetogens living in close proximity to the hydrogen-producing organism [38]. Here, we demonstrate that, in addition, *A. woodii* can also play both parts—the fermenting and the hydrogen consuming part—in one cell. This is the closest proximity one can get. We propose to call this novel type of hydrogen cycling that connects an oxidative and reductive metabolic module in one bacterial cell “intracellular syntrophy.”

Acetogenic microorganisms are phylogenetically very divers [41]. Acetogenesis has been found in different phylogenetic clades of the *Bacteria* and has been studied there for the last hundred years, but rather recently acetogenesis was also found in different phyla of the *Archaea*. This is not only based on genomic but also on physiological analyses [42–44]. These archaea are supposed to grow autotrophically on H_2_ + CO_2_ to produce acetate but also on organic substrates. It is postulated that they ferment the organic substrates such as short chain fatty acids to acetate, alcohols and molecular hydrogen [43]. At the same time the WLP can act as electron sink to make the fermentation energetically possible, which makes fermentation independent from a syntrophic partner [44]. Our mutational analyses in *A. woodii* fully support this model.

The coupling of hydrogen-dependent CO_2_ reduction to hydrogen-producing fermentations allows acetogens to grow, for example, in the deep biosphere on substrates that are otherwise inaccessible for energetic reasons. Hydrogen production from fermentation is common in aquatic sediments and hydrogen production and consumption is essential for anaerobic food webs [45]. Hydrogen production from organic substrates also puts a new perspective on the origin of the eukaryotic cell [43]. Many acetogens have just one soluble hydrogenase like *A. woodii* [46], others such as *Moorella thermoacetica* [47], *Thermoanaerobacter kivui* [48] or *Heimdallarchaeota* and *Odinarchaeota* [43] have membrane-bound, ion-translocating hydrogenase activities as hydrogen consuming respiratory enzymes. Their role in hydrogen transfer is still an open question.
